# Hypoglycemia and Alzheimer Disease Risk: The Possible Role of Dasiglucagon

**DOI:** 10.1007/s10571-024-01489-y

**Published:** 2024-07-08

**Authors:** Naif H. Ali, Hayder M. Al-Kuraishy, Ali I. Al-Gareeb, Najah R. Hadi, Abdullah A. Assiri, Mohammed Alrouji, Nermeen N. Welson, Athanasios Alexiou, Marios Papadakis, Gaber El-Saber Batiha

**Affiliations:** 1 Assistant Professor of Neurology, Department of Internal Medicine, Medical College, Najran University, Najran, Kingdom of Saudi Arabia; 2 Department of Clinical Pharmacology and Medicine, College of Medicine, Mustansiriyah University, Baghdad, Iraq; 3 Head of Jabir Ibn, Hayyan Medical University, Al-Ameer Qu./Najaf-Iraq, PO.Box13, Kufa, Iraq; 4 Department of Pharmacology and Therapeutics, Faculty of Medicine, University of Kufa, Kufa, Iraq; 5 Department of Clinical Pharmacy, College of Pharmacy, King Khalid University, Abha, Kingdom of Saudi Arabia; 6 Department of Clinical Laboratory Sciences, College of Applied Medical Sciences, Shaqra University, Shaqra, 11961 Kingdom of Saudi Arabia; 7 Department of Forensic Medicine and Clinical Toxicology, Faculty of Medicine, Beni-Suef University, Beni Suef, 62511 Egypt; 8 Department of Science and Engineering, Novel Global Community Educational Foundation, Hebersham, NSW 2770 Australia; 9 AFNP Med, 1030 Vienna, Austria; 10 Department of Surgery II, University Hospital Witten-Herdecke, Heusnerstrasse 40, University of Witten-Herdecke, 42283 Wuppertal, Germany; 11 Department of Pharmacology and Therapeutics, Faculty of Veterinary Medicine, Damanhour University, Damanhour, AlBeheira, 22511 Egypt; 12 University Centre for Research & Development, Chandigarh University, Punjab, India; 13 Department of Research & Development, Funogen, Athens, Greece

**Keywords:** Alzheimer disease, Glucagon, Dasiglucagon, Dementia

## Abstract

Alzheimer's disease (AD) is a progressive neurodegenerative disease characterized by memory impairment and cognitive dysfunctions. It has been shown that hypoglycemia can adversely affect AD neuropathology. It is well-known that chronic hyperglycemia in type 2 diabetes (T2D) is regarded as a potential risk factor for the development and progression of AD. However, the effect of recurrent hypoglycemia on the pathogenesis of AD was not deeply discussed, and how recurrent hypoglycemia affects AD at cellular and molecular levels was not intensely interpreted by the previous studies. The underlying mechanisms for hypoglycaemia-induced AD are diverse such as endothelial dysfunction, thrombosis, and neuronal injury that causing tau protein hyperphosphorylation and the accumulation of amyloid beta (Aβ) in the brain neurons. Of note, the glucagon hormone, which controls blood glucose, can also regulate the cognitive functions. Glucagon increases blood glucose by antagonizing the metabolic effect of insulin. Therefore, glucagon, through attenuation of hypoglycemia, may prevent AD neuropathology. Glucagon/GLP-1 has been shown to promote synaptogenesis, hippocampal synaptic plasticity, and learning and memory, while attenuating amyloid and tau pathologies. Therefore, activation of glucagon receptors in the brain may reduce AD neuropathology. A recent glucagon receptor agonist dasiglucagon which used in the management of hypoglycemia may be effective in preventing hypoglycemia and AD neuropathology. This review aims to discuss the potential role of dasiglucagon in treating hypoglycemia in AD, and how this drug reduce AD neuropathology.

## Introduction

Alzheimer disease (AD) is characterized by memory and cognitive dysfunctions (Knopman et al. 2021). AD is the commonest type of neurodegenerative disease in populations > 65 years old. However, early-onset AD (EOAD) may start in genetically susceptible subjects below the age of 65. In addition, late-onset AD (LOAD) is mainly appears in subjects above the age of 85 (Yiannopoulou and Papageorgiou 2020). In AD, there are distinct anatomical changes in the brain characterized by cerebral atrophy, mainly of the entorhinal cortex and hippocampus, that are correlated with cognitive impairment (Calabrò et al. 2021). AD was first reported by the psychiatrist Alois Alzheimer in 1906, following an autopsy of 51-year-old women who suffered from cognitive impairment and disorientation. Later on, Alois Alzheimer described specific brain lesions in patients with memory and cognitive impairments that were senile plaques and neurofibrillary tangles (NFTs) (Behl 2023). A century later, AD became the chief cause of dementia, which represents 75% of dementia cases (Cao et al. 2020). AD threatens around 55 million people worldwide and is expected to reach 107 million by 2050 because of greater lifespans. After 65, women are more likely than men to get AD (Pedroza et al. 2022).

It has been shown that the causes of AD are multifactorial and related to genetic and environmental factors (Chávez-Gutiérrez et al. 2020). More than 95% of AD is sporadic, which is related to different etiopathological factors such as type 2 diabetes (T2D), hypertension, dyslipidemia, low education, and other idiopathic factors (Kosenko et al. 2020). Less than 3% of AD is called familial, which is related to different genetic factors involved in AD neuropathology (Tomiyama et al. 2020). Familial AD is often characterized by the development of EOAD compared to LOAD, which develops in sporadic AD (Tomiyama et al. 2020). In familial AD, mutations in the amyloid precursor protein (APP) gene cause an excessive production of amyloid protein beta (Aβ) (Guyon et al. 2020). As well, mutations in the apolipoprotein E4 (ApoE4) gene contribute to AD neuropathology by increasing Aβ accumulation and hindering its clearance in familial AD (Lamoureux et al. 2021; AlAnazi et al. 2023).

AD neuropathology is characterized by extra-neuronal Aβ accumulation (senile plaque) and intra-neuronal deposition of hyperphosphorylated tau protein (NFTs) (Tomiyama et al. 2020; Alsubaie et al. 2022). Aβ is a small peptide composed of 39–43 amino acids derived from APP through the amyloidogenic pathway via gamma secretase (γ-secretase) and beta secretase (β-secretase). However, APP processing through the non-amyloidogenic pathway promotes the generation of a neuroprotective soluble APP alpha (sAPPα) (Ulaganathan and Pitchaimani. 2023). Normally, APP processing occurs mainly through the non-amyloidogenic pathway. However, in aging and AD, the processing of APP is shifting toward the amyloidogenic pathway (Ulaganathan and Pitchaimani 2023; Al-kuraishy et al. 2023c).

Aβ clearance occurs through various mechanisms, including neuronal enzymes such insulin-degrading enzyme (IDE) and neprilysin, as well as by neuronal autophagy, which removes misfolded proteins (Żukowska et al. 2023). Monomeric Aβ is quickly removed from the cerebrospinal fluid to the systemic circulation for metabolism by the liver and excreted by the kidney (Walsh and Selkoe 2020). Though, trimeric Aβ_40-42_ have a higher propensity for aggregation in the extra-neuronal site to form senile plaque (Balmik et al. 2022; Al-Kuraishy et al. 2023b). The extra-neuronal Aβ_42_ has a neurotoxic effect that results in synaptic dysfunction, inhibition of excitatory neurotransmitters, and progressive neuronal deaths (Singh 2020). The senile plaque has a deleterious effect on the cortical synapses, resulting in increasing memory and cognitive dysfunction (Cuestas Torres and Cardenas 2020). Aβ40 is mainly accumulated in the cerebral vasculature, causing the development of cerebral amyloid angiopathy (CAA), which is often detected in 90% of AD patients (Greenberg et al. 2020). Conversely, CAA may be found in the brains of non-AD dementias such as vascular dementia. Therefore, Aβ accumulation in AD is caused by overproduction and diminished clearance of Aβ (Greenberg et al. 2020).

Furthermore, AD neuropathology is linked to NFTs, which are caused by hyperphosphorylated tau protein aggregation rather than Aβ (Sengoku 2020; Alrouji et al. 2023; Ali et al. 2023, 2024). Tau protein is a small peptide, controls the stability of neuronal microtubules, synaptic plasticity, and axonal transport. Hyperphosphorylated tau protein and NFTs are mainly accumulated in the entorhinal cortex, which affects the hippocampus (Moore et al. 2023; Al-kuraishy et al. 2023d). NFT and Aβ deposits activate microglia causing release of pro-inflammatory cytokines and free radicals with subsequent development of neuroinflammation and oxidative stress (Cai et al. 2022). These neuropathological alterations lead to increase the accumulation and decreased clearance of NFTs and Aβ. Despite these findings, AD neuropathology is exceedingly complicated and associated with multiple signaling pathways and cellular changes (Cai et al. 2022).

Despite advances in research, currently there is no curable treatment for AD neuropathology. As a result, the discovery of new therapy modalities is vital in addressing early AD neuropathology, which began decades prior to symptomatic AD (Self and Holtzman 2023). It has been shown that hypoglycaemia can adversely affect AD neuropathology (Chakrabarty et al. 2022; Al-kuraishy et al. 2023a). It is well-known that chronic hyperglycaemia in T2D is regarded as a potential risk factor for the initiation and progression of AD. However, the effect of recurrent hypoglycaemia on the pathogenesis of AD was not fully discussed by the previous studies. In addition, the effect of recurrent hypoglycaemia on AD neuropathology at cellular and molecular levels was not deeply interpreted by the different studies. Importantly, the glucagon hormone, which controls blood glucose, can regulate different brain functions such as memory and cognitive function (Li et al. 2021). Glucagon increases blood glucose, antagonizing the metabolic effect of insulin (Finan et al. 2020). Furthermore, glucagon-like peptide 1 (GLP-1) improves the cognitive function and memory impairment in both animals and humans (Kong et al. 2023; Bi et al. 2023). Thus, modulation of brain glucagon and GLP-1 signalling pathways may attenuate AD neuropathology by preventing the development of hypoglycaemic episodes. Accordingly, activation of brain glucagon receptors by specific agonists may reduce the development and progression of AD. Therefore, this review aims to discuss the potential role of the glucagon receptor agonist dasiglucagon in the management of AD.

### The Pharmacology of Dasiglucagon

Dasiglucagon is an agonist of the glucagon receptor and produces a similar effect as that of endogenous glucagon. Dasiglucagon through activation of liver glucagon receptors, triggers the stimulation of adenylate cyclase and increases cAMP signalling which induces hepatic gluconeogenesis, glycogenolysis and elevation of blood glucose (Pieber et al. 2021). Dasiglucagon was approved by the FDA in 2021, it mainly indicated in the management of severe hypoglycaemia especially in T2D patients. Dasiglucagon is contraindicated in patients with insulinoma, pheochromocytoma, and hypertension (Li et al. 2020). Dasiglucagon increases blood glucose within 90 min after its administration in T1D patients in a dose-dependent manner (Xu et al. 2021). However, it increases blood glucose within 15 min after its administration in healthy subjects. Dasiglucagon reaches the maximum plasma concentration within 20 min, and it has a similar safety profile to that of glucagon, though mild nausea and vomiting may develop following the administration of dasiglucagon (Xu et al. 2021). Dasiglucagon can interact with some medications; it increases the warfarin effect and leads to hypoglycaemia when taken with β-blockers. Different clinical trials conducted in different countries illustrated the similar efficacy and safety of dasiglucagon in treating hypoglycaemia (Li et al. 2020; Pieber et al. 2021; Xu et al. 2021). Dasiglucagon can increase the blood glucose which has a detrimental effect on AD neuropathology. However, appropriate dosing and timely used of dasiglucagon at time may prevent the progression of AD neuropathology.

### Hypoglycaemia and AD Risk

**Fig. 1:**
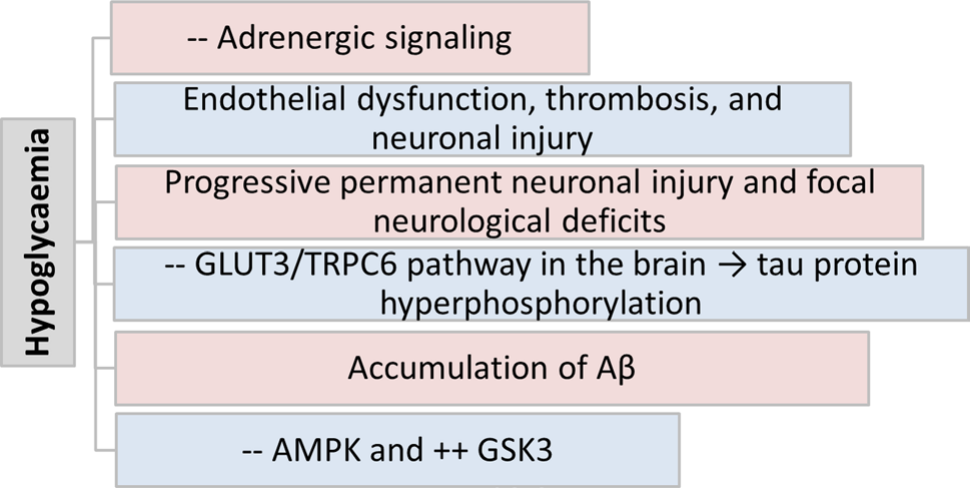
The pathogenesis of AD in relation to effect of hypoglycaemia

It has been shown that both hyperglycaemia and recurrent hypoglycaemia adversely affect the pathogenesis of AD (Chakrabarty et al. 2022). Notably, AD is regarded as type 3 diabetes due the mechanistic interplay between AD and T2D. The brain insulin signalling is highly disturbed in both AD and T2D due to the development of mitochondrial dysfunction, oxidative stress, neuroinflammation, and the accumulation of advanced glycation end products. The accumulation of Aβ is often present in the brains of AD and T2D patients, thus AD is regarded as a metabolic disease caused by the development of insulin resistance (Han et al. 2022). Therefore, recurrent hypoglycaemia in T2D patients may affect AD neuropathology by affecting brain glucose metabolism and brain insulin signalling. Different studies disclosed a closed relationship between recurrent hypoglycaemia and AD (Table 1). Han et al., suggest that recurrent acute hypoglycaemia in elderly T2D patients may lead to the development of dementia due to compromised adrenergic signalling, which counteracts the hypoglycaemic effect on the brain (Han et al. 2022). Of note, a single hypoglycaemic episode can trigger autonomic failure and the development of recurrent hypoglycaemia (Giannakopoulos et al. 2022). A large cohort study followed for 4 years showed that T2D patients with recurrent episodes of hypoglycaemia were at higher risk for dementia and cognitive impairment (Whitmer 2009). T2D patients with more hypoglycaemic episodes were correlated with dementia risk (Whitmer 2009). A population-based study found that recurrent acute hypoglycaemia attacks in newly diagnosed T2D patients increased dementia risk by 16% (Haroon et al. 2015). In addition, a cohort study revealed that hypoglycaemia augments dementia risk in T2D patients. Hypoglycaemic episodes in both diabetic and non-diabetic elderly subjects deteriorate the cognitive function (McNay 2005). A meta-analysis disclosed that recurrent hypoglycaemia in T2D patients is a risk factor for cognitive impairment (Chen et al. 2017). Furthermore, a large population-based cohort study conducted by Han et al. found that recurrent attacks of severe hypoglycaemia increased the risk for the development of AD and vascular dementia through progressive neuronal injury (Han et al. 2022). Many studies highlighted that severe hypoglycaemia deteriorated brain executive function, and even mild hypoglycaemic attacks can provoked focal neurological deficits (Dudley et al. 2022; Verhulst et al. 2022). Originally, it was established that insulin-controlled hyperglycaemia may exacerbate cognitive impairment and AD-like pathology in transgenic mice due to the development of recurrent hypoglycaemic attacks (He et al. 2022). Recurrent hypoglycaemia in streptozotocin-induced diabetes in transgenic mice increases brain neuronal excitability by downregulating glucose transporter 3 (GLUT3), leading to hippocampal mitochondrial dysfunction (He et al. 2022). Supporting to this finding, activation of GLUT3 by transient receptor potential channel 6 (TRPC6) attenuates hypoglycaemia-induced cognitive impairment (He et al. 2020). Dysregulation of TRPC6 expression is involved in the pathogenesis of AD (Prikhodko et al. 2020). Thus, hypoglycaemia-induced cognitive impairment and AD-like pathology are mediated by the inhibition the expression of the GLUT3/TRPC6 pathway in the brain. Conversely, many preclinical studies highlighted that recurrent severe hypoglycaemia may be protective against cognitive impairment in diabetic and non-diabetic rats (McNay and Sherwin 2004). Puente et al. found that mild-moderate hypoglycaemia was protective against brain injury in rats with recurrent hypoglycaemia by improving the paradoxical adaptive response (Puente et al. 2010). Recurrent moderate hypoglycaemia protected against severe hypoglycaemia-induced neuronal damage, limits severe hypoglycaemia-induced neurocognitive dysfunction. This effect is mediated by increasing neuronal threshold for hypoglycaemia-induced seizure through augmentation of brain glycogen content above prehypoglycemic levels which reduce hypoglycaemic neuronal injury (Suh et al. 2007). As well, recurrent moderate hypo glycaemia enhances the inhibitory neurotransmitter, γ-aminobutyric acid (GABA), which limit brain neurotoxicity (Chan et al. 2008). These observations indicated that recurrent attacks of hypoglycaemia increases AD risk by different mechanisms (Fig. 1).

**Table 1:** The link between hypoglycaemia and AD

Study type	Findings	References
A population-based cohort study	Recurrent acute hypoglycaemia increased dementia risk in elderly T2D patients due to compromised adrenergic signalling which counteracts the hypoglycaemic effect on the brain	Han et al. (2022)
A population-based study	Recurrent acute hypoglycaemia attacks in newly diagnosed T2D patients increased dementia risk by 16%	Haroon et al. (2015)
A cohort study	Hypoglycaemic episodes in both diabetic and non-diabetic elderly subjects deteriorate the cognitive function	McNay (2005)
A preclinical study	Insulin-controlled hyperglycaemia exacerbates cognitive impairment and AD-like pathology in transgenic mice due to the development of recurrent hypoglycaemic attacks	He et al. (2022)
A preclinical study	Mild and moderate hypoglycaemia was protective against brain injury in rats with recurrent hypoglycaemia by improving the paradoxical adaptive response	Puente et al. (2010)
A case–control study	Serum AD-related proteins were increased in healthy controls but not in T2D patients with AD following hypoglycaemia suggesting a protective effect of AD-related proteins against the severity of hypoglycaemia	Moin et al. (2021)
Preclinical studies	Severe hypoglycaemia leads to permanent functional and structural brain injury mainly in the hippocampus, frontal lobe, and cerebral cortex	Wright and Frier (2008) and Bree et al. (2009)

The underlying mechanism for the recurrent hypoglycaemia-induced AD and vascular dementia is through the progression of endothelial dysfunction, thrombosis, and neuronal injury (Han et al. 2022). Importantly, recurrent hypoglycaemia enhances the accumulation of aggregated Aβ by increasing the expression of APP mRNA and APP processing toward the amyloidogenic pathway (Ortiz et al. 2022). A preclinical study found that hypoglycaemic episodes reduced the brain expression of AMP-activated protein kinase (AMPK) and increase the expression of glycogen synthase kinase 3 (GSK3). These neuropathological changes increased the accumulation of Aβ and tau protein hyperphosphorylation in the brain (Lee et al. 2013). Interestingly, recurrent hypoglycaemic episodes produced severe brain injury when brain insulin resistance is present (Shpakov et al. 2023). Numerous studies have found that severe hypoglycaemia leads to permanent functional and structural brain injury mainly in the hippocampus, frontal lobe, and cerebral cortex (Wright and Frier 2008; Bree et al. 2009).

**Fig. 2:**
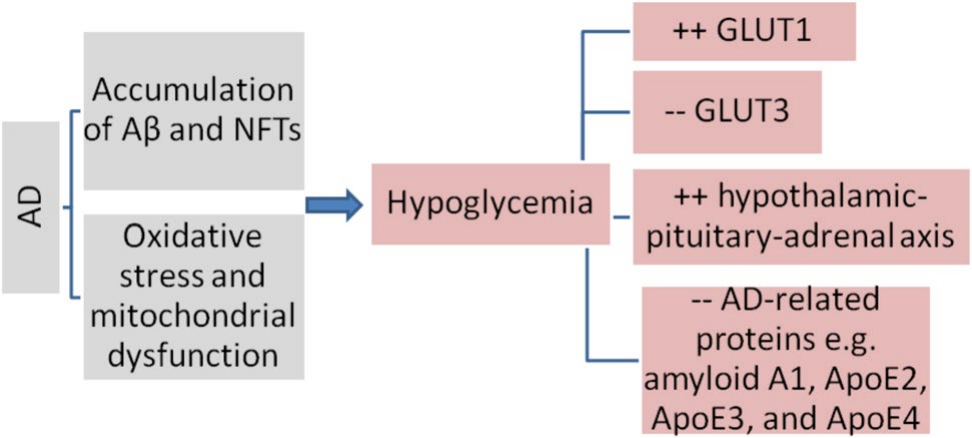
The effect of AD on the development of hypoglycaemia

Therefore, the relationship between hypoglycaemia and AD may be bidirectional; hypoglycaemia triggers AD which experiences recurrent hypoglycaemic attacks. Once the AD neuropathology starts, the hypoglycaemic episodes become more frequent (Biessels 2009). In AD, the brain glucose metabolism is cruelly altered due to the accumulation of Aβ and NFTs which affect insulin signalling and neuronal glucose utilization (Dewanjee et al. 2022). AD neuropathology promotes brain glucose hypometabolism due to oxidative stress and mitochondrial dysfunction (Huang et al. 2023). Therefore, AD brains are more susceptible to the effect of hypoglycaemia which also affects AD neuropathology (Dewanjee et al. 2022). Of interest, prolonged moderate and recurrent hypoglycaemia induces Aβ accumulation and the development of neuroinflammation in transgenic mice (He et al. 2022). In the recurrent hypoglycaemic episodes, GLUT1 which is expressed in the BBB, and GLUT3 which is expressed in the neuron are increased and decreased respectively (He et al. 2022), as a compensatory mechanism to promote brain glucose metabolism (Pitchaimani et al. 2020). A previous experimental study illustrated that the aberrant hypothalamic–pituitary–adrenal axis (HPA) in transgenic mice expressing mutant APP was involved in AD neuropathology by sustaining the brain hypoglycaemic effect (Pedersen et al. 1999). Remarkably, mild hypoglycaemia leads to more effect than severe hypoglycaemia in the induction of AD neuropathology. A case–control study found that serum AD-related proteins such as amyloid ApoE1, ApoE2, ApoE3, and ApoE4 were increased in healthy controls but not in T2D patients with AD following hypoglycaemia (Moin et al. 2021), suggesting a protective effect of AD-related proteins against the severity of hypoglycaemia.The blunted response of AD-related proteins in T2D patients may explain these findings. A large number of AD-related proteins are not diagnostic for AD but can be used in research (Zhao et al. 2019). These verdicts indicated that AD neuropathy mainly amyloid deposits and NFTs, and associated neuroinflammation and oxidative stress augment the development of recurrent hypoglycaemic episodes (Fig. 2).

These findings indicated that recurrent hypoglycaemic episodes are involved the development of AD which predispose for the development and progression of recurrent hypoglycaemic episodes. Therefore, screening of T2D patients for the number of hypoglycaemic episodes is mandatory; also AD patients should be screen for the risk of hypoglycaemic episodes. Hence, early detection and treatment of hypoglycaemic episodes mainly in T2D patients could be preventive measures against the development of AD (Zhao et al. 2019).

### Role of Dasiglucagon in the Management of Hypoglycaemia in AD

Of note, glucagon can cross the BBB and act on the glucagon receptors in the brain, which are abundantly expressed in the brain stem and hypothalamus (Pujadas et al., 2016). The glucagon receptor activates the cAMP/PKA signalling pathway (Wewer Albrechtsen et al. 2023). It has been shown that central administration of glucagon in the experimental animals regulates the peripheral glucose homeostasis (Mighiu et al. 2013). Activation of the brain glucagon receptor improves glucose homeostasis in obese and diabetic animals (Finan et al. 2015). As well, higher serum glucagon is associated with better cognitive function in patients with or without T2D by enhancement of synaptic plasticity and synaptic transmission through cAMP/PKA signalling pathway (Dybjer et al. 2020). High glucagon serum increases the availability of glucose for brain energy under certain conditions, when the BBB permeability is increased due to hyperglycemia or neurodegenerative disease (Janelidze et al., 2017). Glucagon has a neuroprotective effect by increasing the cognitive performance. A population -based study illustrated that glucagon serum level was correlated with better cognitive functions in older people (Dybjer et al. 2020). There are some possible explanations for the association between glucagon and cognitive function, although it is not possible to determine causality in a cross-sectional study. High glucagon may, via systemic hyperglycaemia, increase glucose availability in the brain. Alternatively, glucagon may directly affect neuronal function/synaptic transmission (Dybjer et al. 2020). In relation to AD, an in vitro study demonstrated glucagon inhibits the formation of amyloid fibrils (Gelenter et al. 2019). In addition, glucagon amyloid fibrils can catalyse neuronal amyloid fibrils (Arad et al. 2022) suggesting that glucagon could be effective in the clearance of amyloid peptides in AD. In addition, GLP-1 has a potential role in the regulation of cognitive function and preventing AD neuropathology. Interestingly, GLP-1, GLP-1 analogues and GLP-1 mimetic have neuroprotective effective against AD neuropathology by activating brain GLP-1 receptors mainly in the microglia, neurons and astrocytes (Reich and Hölscher 2022). These agents improve cognitive function, motor and learning function, ameliorating of Aβ pathology, inhibition of neuronal mitochondrial dysfunction and oxidative stress, regulating of brain insulin sensitivity and neuronal autophagy. In addition, GLP-1 improves neurogenesis in AD models by activating the expression of brain-derived neurotrophic factor (BDNF) (Reich and Hölscher 2022). Numerous studies establish that GLP-1 and GIP receptor agonists protect synapses and synaptic transmission from the toxic events that underlie AD. Treatment with GLP-1, GIP, or dual-GLP-1/GIP receptor agonists effectively protected cognition, synaptic trans-mission, long-term potentiation (LTP), and prevented the loss of synapses and neurons in animal models (Hölscher 2018). Findings from preclinical study demonstrated that systemic pre-treatment with exendin-4 can effectively prevent the behavioural impairment induced by neurotoxic Aβ1-42 (Jia et al. 2016) suggesting that the application of exendin-4 or the activation of its signalling pathways may be a talented strategy to mitigate the degenerative processes observed in AD. In addition, liraglutide has a neuroprotective effect by reducing amyloid burden in AD mouse model (Holubová et al. 2019). Importantly, the GLP-1 receptor agonists such as exendin-4 and liraglutide have shown good protective effects in clinical trials in AD patients (Hölscher 2024). Many meta-analyses showed that GLP-1 receptor agonists ameliorate the cognitive ability and memory function in AD models and diabetic patients with AD (Kong et al. 2023; Bi et al. 2023). Hence, both glucagon and GLP-1 have a neuroprotective role against the development and progression of AD (Table 2).

**Table 2:** Effects of glucagon and GLP-1 agonists on AD

Study type	Findings	References
A population-based study	Glucagon serum level is correlated with better cognitive functions in older people	Dybjer et al. (2020)
Preclinical studies	Glucagon inhibits the formation of amyloid fibrils and catalyses neuronal amyloid fibrils	Gelenter et al. (2019) and Arad et al. (2022)
A preclinical study	GLP-1 mimetic has neuroprotective effective against AD neuropathology by activating brain GLP-1 receptors mainly in the microglia, neurons and astrocytes	Reich and Hölscher (2022)
A preclinical study	GLP-1 improves neurogenesis in AD models by activating the expression of brain-derived neurotrophic factor (BDNF)	Reich and Hölscher, (2022)
A preclinical study	GLP-1, GIP, or dual-GLP-1/GIP receptor agonists effectively protected cognition, synaptic trans-mission, LTP and prevented the loss of synapses	Hölscher (2018)
A preclinical study	Exendin-4 can effectively prevent the behavioural impairment induced by neurotoxic Aβ1-42	Jia et al. (2016)
A preclinical study	Liraglutide has a neuroprotective effect by reducing amyloid burden in AD mouse model	Holubová et al. (2019)
Clinical trials	Exendin-4 and liraglutide improve cognitive function in AD patients	Hölscher (2024)
Meta-analyses	GLP-1 receptor agonists ameliorate the cognitive ability and memory function in AD models and diabetic patients with AD	Kong et al. (2023) and Bi et al. (2023)

In this sense, dasiglucagon, through activation of the brain glucagon receptor, may improve cognitive function and reduce AD neuropathology (Ferreira 2021). Interestingly, dasiglucagon is very effective in the management of hypoglycaemia within 10 min in T1D patients (Li et al. 2020). Story and Wilson found that dasiglucagon is more effective than glucagon in the management of hypoglycaemia in terms of efficacy and stability (Story and Wilson 2022). A preclinical finding illustrated that triple receptor agonists of GLP-1, GIP, and the glucagon receptor attenuate AD neuropathology in transgenic mice with mutant APP by reducing the formation of Aβ (Tai et al. 2018). Indeed, dasiglucagon increases hepatic cAMP during the induction of glycogenolysis and gluconeogenesis (Pieber et al. 2021). This effect may also occur in the brain through the activation of the neuronal glucagon receptor. It has been stated that cAMP is an endogenous modulator of Aβ in neuronal N2a cells expressing mutant APP (Canepa et al. 2013). Gopalakrishna et al. illustrated that cAMP-elevating agents attenuate Aβ-induced neurotoxicity (Gopalakrishna et al. 2023). Recently, it has been shown that glucagon receptor agonists are effective in the treatment of obesity and T2D by increasing energy expenditure and inhibiting food intake, despite their hyperglycaemic effects (Novikoff and Müller 2023). Therefore, glucagon receptor agonists could be an effective therapeutic strategy against obesity and T2D that are risk factors for AD development (Al-Kuraishy et al. 2023a). In addition, glucagon receptor agonists enhance the hepatic synthesis of fibroblast growth factor 21 (FGF21) (Kim et al. 2018). FGF21 has a neuroprotective effect against the pathogenesis of AD by regulating autophagy and inflammatory reactions (Kim et al. 2018). Chen et al., found that FGF21 reduced the the development of neurodegeneration in the AD model by regulating various inflammatory signalling pathways such as MAPK, hypoxia-inducible factor 1α, and protein phosphatase 2A (Chen et al. 2019). Therefore, dasiglucagon may improve AD neuropathology by increasing the expression of FGF21 mRNA and neuronal cAMP. These observations indicated that dasiglucagon has a potential neuroprotective effect against AD neuropathology (Table 3).

**Table 3:** Effect of dasiglucagon on AD

Study type	Findings	References
A preclinical study	Triple receptor agonists of GLP-1, GIP, and the glucagon receptor attenuate AD neuropathology in transgenic mice with mutant APP by reducing the formation of Aβ	Tai et al. (2018)
Preclinical studies	Dasiglucagon increases cAMP which attenuate Aβ-induced neurotoxicity	Pieber et al. (2021) and Gopalakrishna et al. (2023)
Preclinical studies	Glucagon receptor agonists enhance the hepatic synthesis of FGF21) which has a neuroprotective effect against the pathogenesis of AD by regulating autophagy and inflammatory reactions	Kim et al. (2018) and Kim et al. (2018)

Furthermore, appropriate use of antidiabetic drugs is essential in T2D patients to prevent the development of hyperglycaemia and hypoglycaemia which known risk factors in the development and progression of AD. Dasiglucagon should be used only in AD and T2D with severe hypoglycaemia, though low therapeutic dosage of dasiglucagon that not affect the blood glucose may be a novel therapeutic strategy in the management of AD by enhancing the synaptic plasticity. Despite these findings, the direct effect of dasiglucagon on AD neuropathology was not evaluated in preclinical and clinical studies. Therefore, this review excites the researchers for further different studies concerning the effect of dasiglucagon on the cognitive function and AD risk.

## Conclusions

AD is the most frequent cause of dementia characterized by memory impairment and cognitive dysfunctions. Chronic hyperglycaemia in T2D is regarded as a potential risk factor for the development and progression of AD. However, hypoglycaemia can adversely affect AD neuropathology. Recurrent hypoglycaemia episodes are the potential risk factor for the development of AD and other types of dementias. Thus, screening of T2D patients for the number of hypoglycaemic episodes is mandatory. Hence, early detection and treatment of hypoglycaemic episodes mainly in T2D patients could be preventive measures against the development of AD. Dasiglucagon is a glucagon receptor agonist and produces a similar effect as that of endogenous glucagon. Dasiglucagon triggers the stimulation of adenylate cyclase and increases cAMP which induces hepatic gluconeogenesis and glycogenolysis and results in the elevation of blood glucose. Dasiglucagon may improve AD neuropathology by increasing the expression of FGF21 mRNA and neuronal cAMP. Despite these findings, the direct effect of dasiglucagon on AD neuropathology was not evaluated in preclinical and clinical studies. Therefore, preclinical and clinical studies are warranted in this setting.

## Data Availability

Data sharing is not applicable to this article as no datasets were generated or analyzed during the current study.
